# Advances in Research on the Activity Evaluation, Mechanism and Structure-Activity Relationships of Natural Antioxidant Peptides

**DOI:** 10.3390/antiox13040479

**Published:** 2024-04-17

**Authors:** Baoting Xu, Qin Dong, Changxia Yu, Hongyu Chen, Yan Zhao, Baosheng Zhang, Panling Yu, Mingjie Chen

**Affiliations:** 1 Institute of Edible Fungi, Shanghai Academy of Agricultural Sciences, Shanghai 201403, China; xbting0919@163.com (B.X.); dongqin@saas.sh.cn (Q.D.); yuchangxia@saas.sh.cn (C.Y.); hychen@saas.sh.cn (H.C.); bsjiayou0912@163.com (B.Z.); yupanling13@163.com (P.Y.); 2 College of Food Science and Technology, Shanghai Ocean University, Shanghai 201306, China; 3 Shanghai Fanshun Edible Fungus Professional Cooperative, Shanghai 201317, China

**Keywords:** natural antioxidant peptides, activity evaluation, antioxidant mechanism, structure-activity relationship, biological effects, redox signaling pathway

## Abstract

Antioxidant peptides are a class of biologically active peptides with low molecular weights and stable antioxidant properties that are isolated from proteins. In this review, the progress in research on the activity evaluation, action mechanism, and structure-activity relationships of natural antioxidant peptides are summarized. The methods used to evaluate antioxidant activity are mainly classified into three categories: in vitro chemical, in vitro cellular, and in vivo animal methods. Also, the biological effects produced by these three methods are listed: the scavenging of free radicals, chelation of metal ions, inhibition of lipid peroxidation, inhibition of oxidative enzyme activities, and activation of antioxidant enzymes and non-enzymatic systems. The antioxidant effects of natural peptides primarily consist of the regulation of redox signaling pathways, which includes activation of the Nrf2 pathway and the inhibition of the NF-κB pathway. The structure-activity relationships of the antioxidant peptides are investigated, including the effects of peptide molecular weight, amino acid composition and sequence, and secondary structure on antioxidant activity. In addition, four computer-assisted methods (molecular docking, molecular dynamics simulation, quantum chemical calculations, and the determination of quantitative structure-activity relationships) for analyzing the structure-activity effects of natural peptides are summarized. Thus, this review lays a theoretical foundation for the development of new antioxidants, nutraceuticals, and cosmetics.

## 1. Introduction

**Figure 1:**
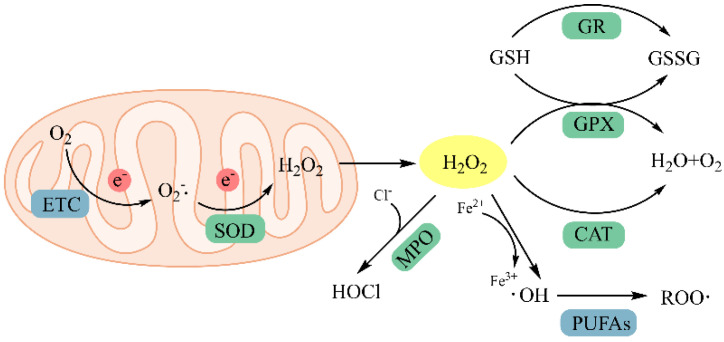
ROS production and metabolic pathways. ETC: respiratory chain; SOD: superoxide dismutase; GSH: reduced glutathione; GSSG: oxidized glutathione; GR: glutathione reductase; GPX: glutathione peroxidase; CAT: catalase; MPO: myeloperoxidase; PUFA: polyunsaturated fatty acid.

Reactive oxygen species (ROS) are mostly byproducts of molecular oxygen escaping from the electron transport chain during mitochondrial oxidative phosphorylation and are characterized by unpaired electrons of oxygen. They mainly consist of free radicals, such as superoxide anions (O_2_^•−^, hydroxyl radicals (^•^OH), alkyl peroxy radicals (ROO^•^), and nonradical substances, such as monoline oxygen (^1^O_2_), hypochlorous acid (HOCl), ozone (O_3_), hydrogen peroxide (H_2_O_2_), and peroxynitrite (ONOO-) [1]. In previous studies, the role of ROS in regulating various physiological functions, including signal transduction, vascular regulation, immune function, and gene activation, has been widely discussed. However, the presence of unpaired electrons in the outermost orbitals causes ROS to be extremely unstable in nature and very susceptible to conversion. Mitochondria are the main source of ROS. The superoxide anion is the first step in ROS production, which is a byproduct of electron transfer in the respiratory chain. Once produced, the superoxide anion is metabolized by a variety of enzyme systems. The first step is its metabolization by superoxide dismutase in the mitochondria into a more diffusible compound, hydrogen peroxide, which diffuses into the cytoplasm and is then metabolized by the antioxidant defense system. However, when there is an excess of ROS in the cell such that the antioxidant defense system is insufficient for resistance, the excess free radicals can have a toxic effect. For example, hydrogen peroxide participates in the Fenton reaction, producing the most harmful free radical, a hydroxyl group (Figure 1). Under normal physiological conditions, intracellular ROS levels remain in equilibrium with endogenous antioxidants to prevent cellular damage. When this critical balance is disrupted, oxidative stress occurs, resulting in the accumulation of excess ROS in the body, which triggers lipid oxidation, DNA/RNA damage, and metabolic imbalance [2]. Therefore, although ROS perform a regulatory role, the oxidative damage produced by excess ROS is related to the pathogenesis of aging, cancer, diabetes, atherosclerosis, neurodegenerative diseases, rheumatoid arthritis, ischemia-reperfusion, obstructive sleep apnea, and other diseases [3]. Moreover, lipid peroxidation induced by ROS can lead to lipid degradation in food or cosmetic matrices, affecting the flavor of food and the efficacy of cosmetics. Therefore, the intake of sufficient antioxidants to prevent or slow the oxidative damage caused by ROS is very important for maintaining normal physiological function and reducing the occurrence of disease. However, commonly used synthetic antioxidants, such as tert-butylhydroquinone (TBHQ), butylhydroxyanisole (BHA), 2,6-di-tert-butyl-p-cresol toluene (BHT), and propyl gallate (PG), exhibit toxicity and carcinogenic effects. Therefore, antioxidants extracted from natural substances, such as polyphenols [4], polysaccharides [5], and peptides [6], which exhibit strong antioxidant properties. In the research of natural antioxidants, antioxidant peptides have become one of the hot directions in recent years.

Antioxidant peptides, which usually consist of 2–10 amino acid subunits and have a molecular weight less than 1 kDa, are characterized by a low molecular weight, low cost, high activity, and easy absorption. Natural antioxidant peptides are relatively safe and healthy compounds that can be isolated and purified from their source materials and are widely used in the food, pharmaceutical, and beauty care industries. To date, many studies have been devoted to the preparation, purification, and characterization of natural antioxidant peptides, and a comparatively complete set of preparation processes has been established. The preparation of natural antioxidant peptides has been summarized in a large number of articles. However, how does one judge their antioxidant activity? After this activity is confirmed, how do these peptides exert their antioxidant effects? Once the mechanism of antioxidant activity has been determined, we need to determine which part of the peptide structure is responsible. Natural antioxidant peptides generally have complex structures and principles of action, and it is difficult to establish uniform activity evaluation indices; as a result, the ability to compare results from various studies is limited. Moreover, the relationship between peptide structure and antioxidant activity has not been well established or elucidated, and the mechanism through which these peptides exert antioxidant effects has not been determined. With respect to these three issues, this paper first summarizes the research on the activity evaluation methods for and potential action mechanisms of natural antioxidant peptides. Then, the relationship between peptide structure and activity is discussed to clarify and predict the structure-activity relationship and mechanism of action so as to improve the overall knowledge of antioxidant peptides isolated from nature and support the production of more-efficient synthetic antioxidant peptides.

## 2. Sources and Preparation of Natural Antioxidant Peptides

**Figure 2:**
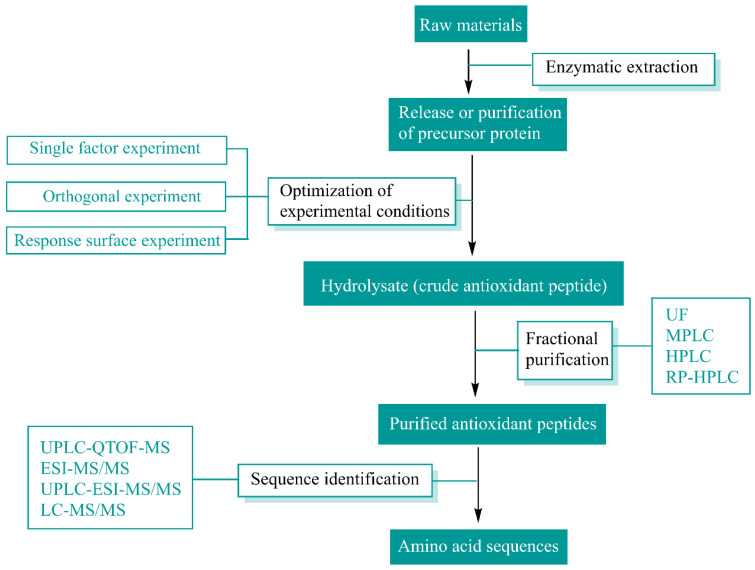
Preparation of natural antioxidant peptides via enzymatic hydrolysis. UF: ultrafiltration; MPLC: medium-pressure liquid chromatography; HPLC: high-performance liquid chromatography; RP-HPLC: reverse-phase high-performance liquid chromatography; UPLC-QTOF-MS: ultrahigh-performance liquid chromatography tandem quadrupole time-of-flight mass spectrometry; ESI-MS/MS: electrospray ionization mass spectrometry; UPLC-ESI-MS/MS: ultrahigh-performance liquid chromatography; LC-MS/MS: liquid chromatography tandem mass spectrometry.

Enzymatic hydrolysis, for which the reaction conditions are mild and controllable, is the most common and relatively safe method for extracting antioxidant peptides from natural substances; when using this approach, the peptides can be stabilized, and the results are reproducible. Enzymes for hydrolyzing antioxidant peptides are primarily categorized as animal proteases, plant proteases, and microbial proteases. Pepsin and trypsin are commonly utilized animal proteases, while papain and pineapple protease are frequently used among plant proteases. Alkaline proteases and neutral proteases are microbial enzymes, which are also commonly employed in the preparation of antioxidant peptides. Techniques such as membrane separation (ultrafiltration and nanofiltration), chromatography (ion exchange chromatography (IEXC), affinity chromatography (AC), gel filtration chromatography (SEC), reversed-phase high-performance liquid chromatography (RP-HPLC)), SDS-polyacrylamide gel electrophoresis (SDS-PAGE), and capillary electrophoresis (CE) are widely used for protein separation and purification. Mass spectrometry (MS) is extensively used for peptide sequence identification due to its high efficiency, sensitivity, and reproducibility. The main types of mass spectrometry include fast atom bombardment, electrospray ionization (ESI), and matrix-assisted laser desorption ionization time-of-flight (MAL-DI-TOF) mass spectrometry. Additionally, liquid chromatography (LC) coupled with MS/MS is widely employed for peptide sequence analysis, with examples including LC-ESI-MS/MS, LC-MAL-DI-TOF-MS/MS, and LC-FTICR (Fourier transform ion cyclotron resonance)-MS/MS. Based on many experimental studies, a series of methods for the enzymatic hydrolysis, separation, purification, and identification of natural antioxidant peptides have been developed, and the specific processes involved are summarized in Figure 2. The purification and identification methods for various antioxidant peptides are summarized in Table 1. Antioxidant peptides derived from natural proteins, such as cereals, legumes, meat, eggs, milk, and marine organisms (marine plants, animals, and bacteria), via enzymatic hydrolysis have been widely reported [3]. Zhao et al. [7] sequentially hydrolyzed purple wheat bran using alkaline enzyme protease to generate antioxidant peptides, fractionated the protein hydrolysis products of purple wheat bran using sephadexed-G25 chromatography and ion-exchange chromatography, and characterized the structures of antioxidant peptides using LC-MS/MS. Kusumah et al. [8] extracted *Vigna radiata* protein, enzymatically hydrolyzed the protein fraction with gastrointestinal enzymes (pepsin and trypsin) or thermolysin, and subsequently sequenced the peptides in the hydrolyzed products. Chen et al. [9] purified antioxidant peptides from chicken breast myofibrillar protein hydrolysate through ultrafiltration and gel filtration chromatography. Wali et al. [10] isolated and purified antioxidant peptides from Bactrian camel milk hydrolysate, demonstrating that peptide fractions from tryptic digests exhibit potent antioxidant activity. Zhang et al. [11] optimized the enzymatic digestion, purification, and identification of antioxidant peptides from egg white. Hu et al. [12] purified and identified antioxidant peptides from *Decapterus maruadsi* hydrolysate through sequential chromatography and electrospray ionization mass spectrometry.

Over the past few decades, there has been a considerable increase in the number of investigations of other sources as well. For example, antioxidant peptides can be obtained from fungal proteins. Aursuwanna et al. [13] prepared peptides from Ganoderma lucidum protein hydrolysate using different proteolytic enzymes, and new peptides with different antioxidant properties, i.e., DRVSIYGWG and ALLSISSF, were discovered. Song et al. [14] identified 25 novel antioxidant peptides with scores above 5 from *Agrocybe aegerita*. As another example, many agro-industrial byproduct inputs have been utilized to prepare natural antioxidant peptides. Wen et al. [15] prepared five antioxidant peptides from watermelon seeds using ultrasonic-pretreatment-assisted enzymatic hydrolysis, among which P1 (RDPEER) possessed the highest free-radical-scavenging and oxygen radical absorbance capacity.

**Table 1:** A variety of antioxidant peptides prepared using the enzymatic hydrolysis method.

Source	Types of Enzymes	Methods of Purification	Methods of Identification	Peptide/Protein Hydrolysate	Reference
Purple wheat bran	Alkaline enzyme protease	sephadexed-G25 chromatography and ion-exchange chromatography	LC-MS/MS	CGFPGHC, QAC, RNF, SSC, and WF	[7]
*Vigna radiata*	Pepsin and pancreatin or thermolysin	SDS-PAGE and Native Blue-PAGE	HPLC-ESI-MS/MS	Low-molecular-weight peptides (3–30 kDa)	[8]
Chicken breast	Pepsin	Membrane ultrafiltration and gel filtration chromatography	LC-ESI-MS/MS	ITTNPYDY, IGWSPLGSL, ITTNPYDYHY, and LRV APEEHPTL	[9]
Bactrian camel milk	Trypsin	Ultrafiltration, gel filtration chromatography (HW-55F), and RP-HPLC	MALDI-TOF-MS/MS	RLDGQGRPRVWLGR, TPDNIDIWLGGIAEPQVKR and VAYSDDGENWTEYRDQGAVEGK	[10]
Egg white	Neutral protease	Ultrafiltration and gel filtration chromatography	UPLC-ESI-MS/MS	LAPYK, SVIRW and PKSVIRW	[11]
*Decapterus* *maruadsi*	Neutrase and trypsin	Ultrafiltration, gel filtration chromatography, and RP-HPLC	LC-MS/MS	KGFR	[12]
*Agrocybe* *aegerita*	Neutral protease	Ultrafiltration and HPLC	MS/MS	Low molecular weight peptides (<3 kDa), 3–10 kDa and >10 kDa)	[14]

## 3. Measurement Methods and Techniques for Determining Antioxidant Activity

Methods for assessing antioxidant activity have significantly advanced over the past few decades. These approaches can generally be categorized into spectroscopic, chromatographic, and electrochemical techniques. The assays used to measure antioxidant activity are classified based on the principle of the chemical reaction involved, which primarily occurs through hydrogen atom transfer (HAT) and electronic transfer (ET) pathways. However, it is important to note that these assays cannot be used in isolation. To gain a comprehensive understanding of antioxidant capacity, multiple methods are often employed. These assays are summarized in Table 2.

### 3.1. Assays Based on HAT

Most tests based on the HAT mechanism involve monitoring the kinetics of competitive reactions followed by quantitative analysis according to the kinetic curve. These tests are utilized to measure an antioxidant’s ability to remove free radicals by providing hydrogen atoms. The underlying principle is as follows:

ROO^•^ + PH/AH→ROOH + P/A

where PH functions as an oxidizing agent or a probe, while AH acts as an antioxidant (referred to as an antioxidant peptide in this article).

In this competitive assay, the antioxidant (AH) undergoes hydrogen atom (H) transfer with ROO^•^ at a faster rate compared to the oxidizing agent or probe (PH). Consequently, the presence of AH inhibits or delays the oxidation of PH induced by ROO^•^, as evidenced by a significantly lower oxidation rate of PH in the system with AH before the complete consumption of ROO^•^ when compared to the control system without AH [16]. The determination methods based on the HAT mechanism primarily include the oxygen free radical absorption capacity (ORAC), total free radical capture antioxidation parameters (TRAP), and crocin bleaching assays (CBA). The ORAC method quantifies an antioxidant’s ability to break down free radicals via measuring its inhibition of ROO^•^ oxidation. In food and biological systems, ROO^•^ is the primary free radical involved in lipid oxidation. Therefore, the ORAC value serves as a biologically relevant reference for assessing antioxidant effectiveness, typically represented by the trolox equivalent [17]. The TRAP and CBA assays share identical principles and common features with the ORAC assay, as they also involve monitoring the kinetic profiles of fluorescence or absorbance decay to evaluate antioxidant activity and express results as trolox equivalents. These high-throughput automation methods are suitable for enzyme-labeling instruments. However, these three methods have limitations due to potential interference from natural pigments and fluorophores present in samples.

### 3.2. Assays Based on ET

Detection based on the ET mechanism involves a redox reaction with an oxidant, which also serves as a probe for monitoring the reaction. This method measures the ability of antioxidants to provide electrons. The principle can be summarized as follows:

M (n) + e (from AH) → M (n − 1) + AH^+^

The oxidant M captures electrons from AH, resulting in a color change of M. The degree of color change is directly proportional to the concentration of AH. The cessation of a color change ceases indicates that the end point of the reaction has been reached and that the degree of color change corresponds to the concentration of AH [16]. Popular methods for assessing antioxidant activity involve their ability to act as electron donors and reduce non-free radicals, primarily various metal ions. These methods include the ferric reducing antioxidant capacity (FRAP) assay and the copper reduction antioxidant capacity (CRAC) assay [18]. In the FRAP test, antioxidants reduce ferric ion-TPTZ complexes, leading to a strong color reaction where Fe^3+^ is converted into Fe^2+^. Therefore, oxidation resistance can be determined by measuring absorbance difference at 593 nm compared to standard values. It should be noted that this test is conducted under acidic conditions (pH = 3.6) in order to dissolve iron effectively. Although this method exhibits high sensitivity, its reaction time may vary depending on the substrates involved; hence, it should be used in conjunction with other assays if necessary. The CRAC assay shares similarities with the FRAP assay except that Cu replaces Fe; during this process, Cu^2+^ is reduced to Cu^+^, and absorbance is measured at 450 nm instead [19].

### 3.3. Mixed-Mode Assays (HAT/ET)

The 2,2′-azodiiso-3-ethylbenzazoline-6-sulfonic acid (ABTS) and 2,2-diphenyl-1-trinitrohydrazine (DPPH) free radical assays are widely employed methods for evaluating antioxidant activity, assessing the ability of antioxidants to act as electron donors in neutralizing free radical chromophores (such as ABTS^•+^ and DPPH^•^) or fluorophores. The mechanisms of HAT, ET, and proton-coupled electron transfer (PCET) play varying roles under different reaction conditions, such as pH and solvent type, leading to distinct outcomes [20]. The ABTS assay measures the extent of ABTS free radical neutralization by antioxidants, where the presence of antioxidants results in reduced absorption at 734 nm, leading to a lighter-color solution. On the other hand, the DPPH assay evaluates how antioxidants neutralize DPPH free radicals; upon neutralization, a purple-colored DPPH solution becomes yellow with absorbance measured at 515 nm. While both hydrophilic and lipophilic systems can be assessed using the ABTS method, only hydrophobic systems are suitable for analysis with DPPH [19]. However, it is important to note that neither the DPPH nor ABTS radicals used in these assays exist in biological systems; hence, these methods lack direct biological relevance. Additionally, performing the ABTS assay requires neutral conditions, while pH sensitivity affects the outcome of the DPPH assay, and organic solvents are necessary for dissolving DPPH radicals.

**Table 2:** Different techniques used to measure antioxidant properties.

Techniques	Mechanism	Assay Method	Advantages	Limitations
Spectrometry	HAT	ORAC	Biologically significant and suitable for high-throughput assays.	The presence of natural pigments and fluorophores in the samples may interfere with the absorbance and fluorescence readings.
		TRAP		
		CBA		
	ET	FRAP	Analysis is simple, rapid, cost-effective, and does not require special equipment.	To maintain iron solubility and drive electron transfer, experiments are performed under acidic conditions (pH 3.6); Prussian blue tends to precipitate and stain the assay tubes; and it is a non-radical method that correlates poorly with other antioxidant activity measurements [17].
		CRAC	In contrast to the FRAP method, this assay requires a low redox potential to be achieved and is suitable for both lipophilic and hydrophilic systems; the reagents are more stable and readily available; it is to some extent less demanding in terms of reaction conditions (e.g., temperature, humidity, and pH); and it meets the requirements for evaluating the antioxidant capacity of endogenous and dietary molecules in vitro [20].	Oxidative damage inflicted on biomolecules may be involved.
	HAT/ET	DPPH	Low cost, reproducibility, room temperature suitability, and automation possibilities.	Sensitivity is affected by a variety of factors, such as the type of solvent used, the presence of hydrogen and metal ions, and the freshness of the DPPH reagent; there are many compounds with overlapping absorption spectra in the same wavelength range as DPPH.
		ABTS	Inexpensive and simple to perform; wide range of applicability, and many antioxidants are suitable; it is suitable for both hydrophilic and lipophilic systems.	Lack of biological relevance.
Chromatography	Adsorption and partition chromatography	GC	High sensitivity; wide detection range, i.e., a variety of detection methods can be used; simple process; and low cost.	The sample must be thermally stable and non-decomposable; sample processing is complicated and requires a certain level of skill.
	Adsorption, partitioning, size exclusion, ion exchange and affinity chromatography	HPLC	High accuracy; sample preparation is simple; and a variety of detection methods can be used.	Higher column cost and susceptibility to contamination; relatively slow separation speed; and requires special operation and maintenance.
Electrochemistry	Mass transport to the electrode	Cyclic voltammetry	Adequate analysis of the body’s antioxidant defense system; simple instrumentation and operation; cost-effective and efficient; can be easily applied in clinical laboratories [21].	The determination of antioxidant activity via cyclic voltammetry may be disrupted by other forms of non-antioxidant reducing matter such as reducing sugars.

## 4. Activity Evaluation Methods for Natural Antioxidant Peptides

Until now, the overall antioxidant activity of crude proteins and purified peptides obtained enzymatically from natural proteins has not been characterized using exclusive methods. Different chemical tests combined with highly sensitive and automated detection techniques have been employed to evaluate the antioxidant activity of bioactive peptides. Oxidation systems have also been extended from chemical substrates to food models, cell lines, and living tissues [19]. Therefore, the antioxidant activity of natural peptides is measured through three main methods, namely, in vitro chemical, in vitro cellular, and in vivo animal assays, each of which exhibits different antioxidant effects.

### 4.1. In Vitro Chemical Methods and Their Effects

In vitro chemical methods are convenient, rapid, and highly reproducible and automated; therefore, these methods are widely used in the screening and preliminary evaluation of natural antioxidant peptides. The in vitro chemical methods can be summarized according to the three biological effects induced by each chemically assessed assay: the scavenging of free radicals, chelation of transition metals, and inhibition of lipid peroxidation reactions [22].

#### 4.1.1. Scavenging Free Radicals

The methods based on scavenging free radicals constitute the ORAC, TRAP, CBA, CRAC, DPPH, and ABTS assays. Fontoura et al. [23] confirmed the antioxidant activity of LPGPILSSFPQ, a peptide obtained from feather hydrolysate, by detecting ABTS and TRAP. Xiao et al. [24] purified two peptides, YYCQ and RWGG, from chicken hydrolysate, and YYCQ was the most active peptide according to ORAC and ABTS assays. Tonolo et al. [25] tested six kinds of milk-derived peptides by scavenging DPPH and ABTS free radicals and CBA, and the results showed that the peptides YVPR and VPYPQR exhibited good antioxidant activity. Using the DPPH, ABTS radical scavenging, and FRAP assays, Hu et al. [26] isolated three novel antioxidant peptides, LSPGEL, VYFDR, and PGPTY, from *Gracilaria lemaneiformis* protein hydrolysate and purified them.

There are a variety of ROS in the human body, and some free radicals, such as O_2_^•−^ and ^•^OH, are scavenged by mechanisms outside of the above two categories. The O_2_^•−^ scavenging test is positively correlated with results obtained from the ABTS, DPPH, and FRAP assays, while the ^•^OH radical scavenging ability is more strongly correlated with the hydrogen transfer ability of antioxidants [27]. Liu et al. [28] isolated antioxidant peptides from yak casein hydrolysates and evaluated the synthesis of antioxidant peptides with O_2_^•−^ and ^•^OH-scavenging activities (IC_50_ = 0.52 and 0.69 mg/mL): Arg-Glu-Leu-Glu-Glu-Leu.

#### 4.1.2. Chelating Metal Ions

Free radicals may originate from heavy metals and transition metals and can cause diseases associated with oxidative stress. Imbalances in the levels of metals, such as iron, manganese, copper, and zinc, can lead to cardiovascular diseases, cancers, etc., as well as neurodegenerative diseases, such as Alzheimer’s disease and Parkinson’s disease [29]. Iron and copper ions can participate in the formation of ROS in vivo through Fenton and Haber-Weiss reactions (H_2_O_2_ + Fe^2+^/Cu^+^ → Fe^3+^/Cu^2+^ + HO- + ^•^OH), forming products such as O_2_^•−^, H_2_O_2_, and ^•^OH, which are strong oxidizers [30]. Antioxidants can form complexes with metals, preventing metal ions from acting as initiators of lipid oxidation, thus preventing or delaying lipid oxidation induced by metal ions. Therefore, the ability of antioxidant peptides to chelate metal ions has been used as an indicator of activity, normally in combination with other antioxidant assays. However, the results obtained using this method poorly correlate with the FRAP, CRAC, ABTS, and DPPH assays. Ethylenediamine tetraacetic acid (EDTA) is a canonical metal chelator that is used in many food and pharmaceutical applications [17]. In most tests, the EDTA equivalent is used to express the metal-ion-chelating capacity of antioxidants. Zhang et al. [31] identified five new antioxidant peptides from a *Channa Argus* decoction that showed high Fe^2+^-chelating ability, and the Fe^2+^-chelating ability of SDGSNIHFPN (IC_50_ = 4.60 ± 0.05 mM) was significantly greater than that of the control, EDTA (IC_50_ = 1.67 ± 0.05 mM).

#### 4.1.3. Inhibition of Lipid Peroxidation

Lipid peroxidation is a free radical chain reaction that typically involves the following stages: initiation, propagation, and termination. The polyunsaturated fatty acid carbon-carbon double bonds (C=C) present in organisms are susceptible to attack by free radicals, thus causing a chain reaction involving lipid peroxidation and the formation of lipid peroxidation products. Therefore, indicators reflecting the degree of lipid oxidation can be used for evaluating the antioxidant capacity of peptides. Malondialdehyde (MDA), which is used as a marker of lipid peroxidation, is one of the most common lipid peroxidation secondary products. Thiobarbituric-acid-reactive substances (TBARS), ferric thiocyanate (FTC), gas chromatography (CG), high-performance liquid chromatography (HPLC), and electron spin resonance (ESR) are the most common MDA assays [32]. Lipid peroxidation models, such as linoleic acid, low-density lipoprotein, and animal and vegetable fat models, are commonly used to evaluate the ability of antioxidant peptides to inhibit lipid peroxidation. In addition to stimulating lipid oxidation through the Fenton reaction, transition metal ions can breakdown lipid hydroperoxides into more reactive peroxyl and alkoxyl radicals. Therefore, the method used to test for anti-lipid peroxidation effects is often combined with the above two methods to evaluate antioxidant activity. Torres-Fuentes et al. [33] investigated the ability of peptide fractions purified from chickpea protein hydrolysates to inhibit copper-mediated lipid peroxidation in three different lipid substrates (β-carotene, a mixture of unsaturated fatty acids, and low-density lipoprotein) using the TBARS assay. The results showed that the copper-chelated peptide fraction of chickpea plants, which contained the highest level of histidine, exhibited the strongest antioxidant activity. This was mainly because histidine can bind to copper and act as a hydrogen donor through its imidazole ring. The chemical methods for assessing the activity of natural antioxidant peptides from different sources and the action mechanisms of each peptide are summarized in Table 3.

### 4.2. Cell Methods In Vitro and Their Effects

Compared with chemical methods, cell models can reveal the effects of natural antioxidant peptides under physiological conditions more comprehensively. Since animal models and human clinical trials are expensive, time consuming, and complex, cellular antioxidant testing is an effective tool for initially evaluating or screening target natural antioxidant peptides before conducting in vivo experiments. The protective effect of natural antioxidant peptides on damaged cells may further reflect the mechanism by which peptides exert protective effects against oxidative stress.

#### 4.2.1. Establishment and Evaluation of the Cell Model

**Figure 3:**
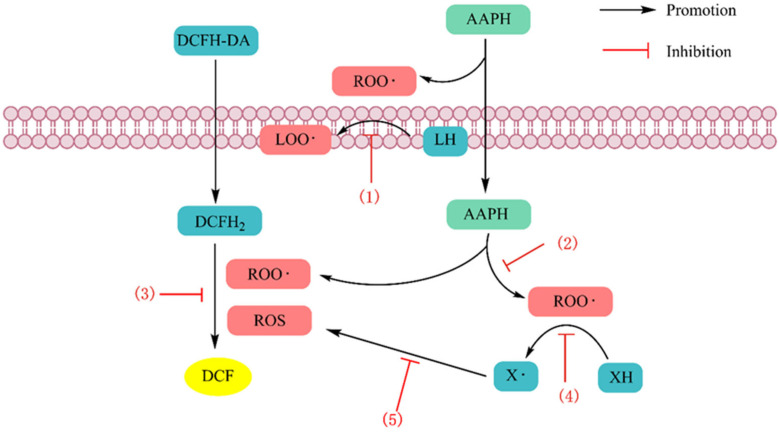
Schematic diagram of the cell antioxidant activity assay (CAA). DCFH-DA: 2′,7′-dichlorofluorescin diacetate; DCFH: 2′,7′-dichlorodihydrofluorescein DCF: 2′,7′-dichlorofluorescein. DCFH-DA is a universal indicator of oxidative stress; it has cell membrane permeability and does not produce fluorescence. Once in the cell, DCFH is hydrolyzed by cell esterase to produce DCFH, which is then oxidized rapidly to produce the strong fluorescent product DCF. Antioxidant peptides can protect DCFH2 from oxidation into DCF by free radicals through the following mechanisms: (1) scavenging ROO· in the membrane to reduce lipid peroxidation; (2) reacting with AAPH to prevent the formation of ROO; (3) competing with DCFH2 for oxidants; (4) reacting with ROO· to avoid other free radicals; and (5) inhibiting the redox reaction that produces ROS and prevents DCF production.

**Figure 4:**
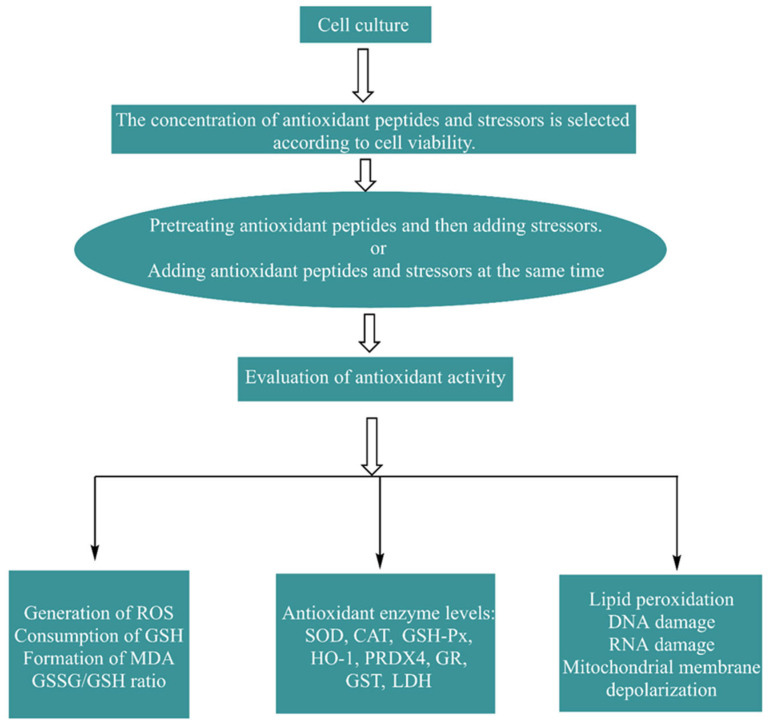
Flow chart of the in vitro cell culture experiment. SOD: superoxide dismutase; CAT: catalase; GSH-Px: glutathione peroxidase; OH-1: heme oxygenase-1; PRDX4: peroxiredoxin 4; GR: glutathione reductase; GST: glutathione S-transferase; LDH: lactate dehydrogenase.

The common mammalian cell lines used to construct oxidative stress models are human epithelial colorectal adenocarcinoma cells (Caco-2), human liver cancer cells (HepG2), human normal hepatocellular carcinoma cells (Chang), cervical adenocarcinoma cells (HeLa), human umbilical vein endothelial cells (HUVECs), human neuroblastoma cells (SH-SY5Y), rat adrenal pheochromocytoma cells (PC12), and mouse macrophages (RAW264.7). Other studies have used isolated cells, such as human erythrocytes, rat hepatocytes, and microbial cells, such as those from *Saccharomyces cerevisiae* strains [37]. The most widely utilized sources of oxidative stress in cellular antioxidant testing are H_2_O_2_, lipophilic tert-butyl hydroperoxide (t-BHP), and 2,2-azobis(2-methylpropylimide) dihydrochloride (AAPH). The cellular antioxidant activity assay (CAA), which measures the ability of antioxidant peptides to quench ROS in cell cultures, is a biologically representative method that involves cellular biochemical processes (e.g., antioxidant uptake, distribution, bioavailability, and metabolism) [38], and the principle of this assay is shown in Figure 3. The 3-(4,5-dimethyl2-thiazolyl)-2,5-diphenyl-2H-tetrazolium bromide (MTT) assay was used to assess the viability of cell models exposed to antioxidant peptides without oxidative stress treatment and verify whether the antioxidant peptides were cytotoxic. The in vitro cellular assay is a biological method that simulates cellular oxygen stress injury and measures multiple oxidative indices to determine antioxidant activity. The evaluation indices in the cell models mainly included the following categories: one category ws the promoting effect of antioxidant peptides with respect to endogenous enzymes (superoxide dismutase (SOD), glutathione reductase (GR), glutathione peroxidase (GSH-Px), glutathione S-transferase (GST), and catalase (CAT)) and nonenzymatic antioxidants (GSH); the second category was the inhibitory effect of antioxidant peptides on oxidative products (ROS, MDA, and GSSG) [9]. The process and evaluation indicators used to construct a cell oxidative stress model are shown in Figure 4. Zhang et al. [39] investigated the protective effect of tilapia antioxidant peptides against AAPH-induced oxidative stress in HepG2 cells. MTT assays showed that all the peptides were nontoxic at concentrations up to 100 μM. The CCA results showed that LPGYF exhibited the highest ROS scavenging ability and that PGY could enhance the activities of CAT and SOD. Tao et al. [40] explored the antioxidant activity of *Moringa oleifera* leaf peptides treated with H_2_O_2_ in HepG2 cells and reported that the peptide with the sequence LALPVYN (with a molecular weight of 44.24 Da) showed the strongest antioxidant activity. The MTT assay suggested that the peptide exerted no toxic effects on the cells at concentrations ranging from 50 to 500 μg/mL. And the CAA assay showed that the peptide could reduce intracellular ROS content. Moreover, this peptide significantly increased CAT, GSH-Px, and SOD enzyme activities and decreased MDA content.

#### 4.2.2. Biological Effects of Antioxidant Peptides in Cells

The effects of natural antioxidant peptides are not limited to chemical antioxidant activities (scavenging free radicals, chelating metal ions, and inhibiting lipid peroxidation): they have also been demonstrated to exhibit cellular antioxidant activities including the inhibition of oxidative enzyme activity and modulation of antioxidant expression. These cellular effects of antioxidant peptides are summarized below and in Table 4 for each category. The activity of natural antioxidant peptides is often not a manifestation of one effect but rather a combination of effects.

##### Inhibition of Related Oxidative Enzymes

Nicotinamide adenine dinucleotide phosphate (NADPH) oxidase (abbreviated NOX), the isoform (NOX2) of which is present in phagocytic cell membranes, is activated in the presence of molecular oxygen (O_2_) and forms an active complex with another membrane subunit, p22phox. This complex catalyzes a sequential reaction that generates ROS (especially O_2_^•−^), resulting in a variety of pathological conditions, such as diabetes, cardiovascular disease, neurodegenerative diseases, aging, and tumor and cancer development and progression [41]. NADPH plays a key role in the cellular antioxidant system by acting as an important cofactor for GR, which converts GSSG to GSH, reducing H_2_O_2_ and other peroxides and inactivating ROS. Fang et al. [42] discovered that hazelnut peptides have a significant effect on protecting HUVECs from oxidative stress damage, and three of these peptides (EW, ADGF, and DWDPK) inhibited NADPH oxidase activity by decreasing the expression of NOX2 and p22phox, which reduced cellular oxidative stress injury. Cottonseed meal is widely found in different fish feeds. Yuan et al. [43] reported that the antioxidant peptide LGSPDVIVIR extracted from cottonseed meal protein hydrolysates significantly downregulated the mRNA expression of NOX2 in fish hepatocytes and increased the antioxidant capacity of the fish.

##### Activation of Endogenous Antioxidant Enzymes and the Nonenzymatic Defense System

The antioxidant defense system consists of antioxidant enzymes (e.g., SOD, CAT, and GSH-Px) and other low-molecular-weight antioxidants (e.g., ascorbic acid, vitamin E, and glutathione). SOD and CAT are two common endogenous antioxidant enzymes that can convert O_2_^•−^ into O_2_ and H_2_O to resist oxidative stress in vivo. As a product of lipid peroxidation, MDA can destroy the structure and function of cells and indirectly reflect the degree of oxidative stress injury. Ma et al. [44] reported that the mechanism by which soybean peptides underwent intracellular antioxidation and detoxification was related to nonenzymatic and enzymatic defense systems. The researchers used AAPH-induced erythrocyte hemolysis to study the cellular antioxidant activity of a 1 kDa soybean peptide. The results showed that the soybean peptide could significantly restore the activities of intracellular SOD, GSH-Px, and CAT; inhibit the production of intracellular MDA; and prevent the consumption of GSH. Li et al. [45] reported that porcine plasma antioxidant peptide (EDEQKFWGK) strengthened the oxidative defense system of HepG2 cells by diminishing the levels of reduced ROS and MDA and increasing the activities of antioxidant enzymes (SOD, CAT, and GSH-Px). The cell membrane is mainly composed of lipid conjugates, especially polyunsaturated fatty acids, and easy to oxidize. Hu et al. [46] reported that low-molecular-weight peptides from grass carp scale antioxidant peptides can increase the potency of the activities of SOD, CAT, and GSH-PX; decrease the levels of ROS and MDA; and reduce the damage to DNA and the cell membrane. Wang et al. [47] studied the cytoprotective effects of the antioxidant peptide from Antarctic krill (*Euphausia superba*) hydrolysate on H_2_O_2_-induced oxidative stress in Chang hepatocytes and identified two peptides, LKPGN and LQP, that could effectively scavenge excessive ROS, increase the content of SOD and GSH-Px, reduce the levels of MDA, and reduce oxidative-damage-induced apoptosis by inhibiting mitochondrial membrane potential.

**Table 4:** Cellular effects of antioxidant peptides from different sources.

Source	Peptide/Protein Hydrolysate	Cell Model	Biological Effect	Reference
Tilapia	LPGYF, PGY	HepG2 cells exposed to AAPH	The peptides not only scavenged intracellular ROS but also enhanced the activity of CAT and SOD, resulting in the activation of the cellular antioxidant defense system.	[39]
*Moringa oleifera* leaves	LALPVYN	HepG2 cells exposed to H_2_O_2_	The peptide scavenged intracellular ROS and also enhanced endogenous antioxidant defenses, including antioxidant enzyme defenses and the glutathione system, resulting in cytoprotection via reducing MDA and ROS production in oxidatively damaged cells.	[40]
Hazelnut	EW, ADGF, DWDPK	HUVECs exposed to Ang II (angiotensin II)	The peptides upregulated the activities of antioxidant enzymes (CAT, SOD, and GSH-Px), downregulated LDH and MDA levels, and inhibited NOX activity and ROS production by decreasing NOX4 and p22phox levels.	[42]
Cottonseed meal	LGSPDVIVIR	Hepatocytes exposed to H_2_O_2_	The peptide increased the potency of SOD and CAT activities and decreased MDA content and downregulated NOX2 mRNA expression to inhibit O_2_^•−^ and ROS production.	[43]
Soybean	Hydrolysis products <1 kDa	Red blood cells exposed to AAPH	The peptides restored the activity of antioxidant enzymes (SOD, GSH-Px and CAT) and inhibited MDA production and GSH depletion	[44]
Porcine plasma	EDEQKFWGK	HepG2 cells exposed to H_2_O_2_	This peptide decreased ROS and MDA content and increased antioxidant enzyme (SOD, CAT, GSH-Px) in order to enhance the oxidative defense system of HepG2 cells.	[45]
Grass carp scales	Low molecular weight peptide of 358–986 Da	HepG2 cells exposed to H_2_O_2_	These peptides generated increases in SOD, CAT, and GPX activities and decreases in ROS levels and MDA content.	[46]
*Euphausia superba*	LKPGN, LQP	Chang liver cells exposed H_2_O_2_	These two peptides increased the levels of antioxidant enzymes (SOD and GSH-PX) to scavenge excess ROS, increased mitochondrial membrane potential, and decreased DNA damage and MDA content.	[47]

### 4.3. In Vivo Animal Methods and Their Effects

The evaluation of chemical antioxidant activity and cellular antioxidant activity has been reviewed previously, and both of these evaluation methods are in vitro evaluation methods. Although the activity of natural antioxidant peptides and potential mechanisms of action can be measured using in vitro experiments, animal experiments are necessary and effective for evaluating the antioxidant capacity and bioavailability of bioactive peptides. However, due to the high costs and long testing cycles needed for animal research and human trials, studies on the role of natural antioxidant peptides in vivo using different animal models are rare. Furthermore, animal experiments can be affected by a range of biological effects caused by antioxidant action in addition to cellular effects. The in vivo effects of various peptides are summarized in Table 5.

According to existing studies, changes in oxidative stress in vivo can also be monitored by measuring oxidative indices when studying the in vivo effects of antioxidant peptides. Among mammals, rats and mice are currently the most common animal models. Han et al. [48] treated mouse livers with t-BHP to establish an oxidative stress model and reported that soluble rice protein hydrolysate increased the activities of antioxidant enzymes (such as CAT and GSH-Px) and the level of GSH and decreased the content of MDA. Tsai et al. [49] reported that potato bioactive peptides activate the Nrf2-dependent antioxidant defense mechanism in rat kidneys, which leads to the activation of the expression of endogenous antioxidant proteins (including SOD1, SOD2, PRDX2, HO-1, and GSH-Px4), thereby modulating the oxidative stress associated with hypertension. In addition, some nonmammals with a short life cycle and the same stress response as humans, such as *Drosophila*, *Caenorhabditis elegans*, and zebrafish, are often used as animal models [27]. Qiu et al. [50] observed that ERJ-CP peptide significantly prolonged the average lifespan of *Drosophila*; increased levels of SOD, GSH-Px, and CAT; and decreased the content of MDA and PCO. Xu et al. [51] revealed that C-phycocyanin peptides (MHLWAAK, MAQAAEYYR, and MDYYFEER) significantly protected zebrafish embryos from H_2_O_2_-induced oxidative damage by inhibiting ROS production, preventing MDA formation, and upregulating SOD and CAT activities. Wang et al. [52] discovered that Yak bone peptides (GASGPMGPR and GLPGPM) significantly prolonged lifespan, enhanced the activities of SOD and CAT, and diminished the content of MDA and ROS in *Caenorhabditis elegans*.

In addition to verifying the antioxidant capacity of bioactive peptides, in vivo studies can explore the effects of antioxidant peptides on a series of biological effects caused by oxidative stress, such as with respect to improving memory and antihypertensive effects and prolonging life. Acetylcholine (ACh), a neurotransmitter, plays an important role in regulating brain cognitive function [53]. Acetylcholinesterase (AChE) can degrade Ach, and an increase in AChE activity may lead to the termination of synaptic transmission. Shu et al. [53] showed that sesame cake protein hydrolysate could increase the activity of GSH-Px and SOD in the hippocampus, decrease the content of MDA and inhibit lipid peroxidation in the hippocampus, and improve memory capacity by increasing AChE activity. Ren et al. [54] reported that the antioxidant hydrolytic peptides (<3 kDa) produced by Manchurian walnut improved memory by inhibiting ROS and enhancing GSH-Px and SOD activities to reduce oxidative stress in mice. These walnut peptides also inhibited the decrease in ACh content induced by scopolamine, ameliorating memory impairment in mice. Hypertension promotes an imbalance in cellular ROS production/clearance, which provides favorable conditions for oxidative stress. In rats, potato bioactive peptides stimulated the Nrf2 antioxidant pathway, which activated the oxidative stress defense system, lowered hypertension, and reduced renal cell apoptosis via the DJ-1 and AKT signaling pathways [49]. Oxidative stress is a key factor in the process of aging. Studies have shown that inhibiting mTOR signaling can extend the lifespan of many species. mTOR signal transduction is a ROS-sensitive pathway, and the antioxidant effect of substances may affect the expression of mTOR. Crimson snapper scale peptides play an antioxidant role and can extend the lifespan of drosophila by blocking mTOR signal transduction and activating autophagy [55]. Cai et al. [55] studied the protective effect of crimson snapper scale peptides and their mechanism of action in *Drosophila melanogaster* under antioxidant stress. The peptide reduced the accumulation of the lipid oxidation end product MDA and the protein carbonylation product PCO in aged *D. melanogaster* by upregulating the expression of antioxidant genes (*SOD1*, *SOD2*, and *CAT*) and enhancing the activities of antioxidant enzymes (SOD, CAT, and GSH-Px). Lin et al. [56] reported that *Stichopus variegates* peptides effectively prolonged the life span of normal *Drosophila* and D-galactose-induced-aging *D. melanogaster* by upregulating the expression of the antiaging factor Klotho, activating SOD and GSH-Px, inhibiting lipid peroxidation and protein oxidation, and alleviating D-galactose-induced oxidative damage in mice.

**Table 5:** Biological effects of antioxidant peptides from different sources in animals.

Source	Peptide/Protein Hydrolysate	Animal Model	Biological Effect	Reference
Rice	Soluble rice protein hydrolysate	Oxidative stress in t-BHP-treated mice	The peptides increased the activities of antioxidant enzymes (CAT, GSH-Px, etc.) and the levels of GSH and reduced the content of MDA; they also inhibited the expression of oxidase NOX4.	[48]
Potatoes	Protein hydrolysate	Spontaneously hypertensive rats	The peptides activated the Nrf2-dependent antioxidant defense mechanism, which prompted the expression of endogenous antioxidant proteins (SOD1, SOD2, PRDX2, HO-1, and GSH-Px4). They also alleviated hypertension through DJ-1 and AKT signaling pathways.	[49]
ERJ-CP	Low-molecular-weight peptides (<1000 Da)	Oxidative stress in H_2_O_2_- treated *Drosophila*	These peptides prolonged the average lifespan of *Drosophila*; increased the levels of SOD, GSH-Px, and CAT; and decreased the content of MDA and PCO.	[50]
C-phycocyanin	MHLWAAK, MAQAAEYYR and MDYYFEER	Oxidative damage in H_2_O_2_- treated zebrafish embryos	These peptides inhibited ROS production, prevented MDA formation, and upregulated SOD and CAT activities	[51]
Yak bones	GASGPMGPR and GLPGPM	Oxidative stress in *C. elegans*	These peptides prolonged lifespan, enhanced the activities of SOD and CAT, and diminished the content of MDA and ROS	[52]
Sesame cake	Protein hydrolysate	Oxidative stress in mice	The peptides increased the activity of GSH-Px and SOD; decreased the activity of MDA and lipid peroxidation; and increased the activity of AChE to improve memory ability.	[53]
Manchurian walnut	Protein hydrolysate (<3 kDa)	Memory impairment in mice treated with scopolamine	The peptides inhibited ROS and increased the activity of GSH-Px and SOD and inhibited the decrease in Ach content.	[54]
Crimson snapper scales	Protein hydrolysate	Oxidative stress in *C. elegans* exposed to paraquat and ultraviolet radiation	The peptides reduced the accumulation of peroxide products (MDA and PCO); enhanced the activities of antioxidant enzymes (SOD, CAT, and GSH-Px); upregulated the expression of antioxidant genes (*SOD1*, *SOD2,* and *CAT*), blocked mTOR signal transduction, and activated autophagy to prolong life.	[55]
*Stichopus variegates*	Protein hydrolysate	*Drosophila* and D-galactose-treated senescent mice	The peptides activated SOD and GSH-Px, inhibited lipid peroxidation and protein oxidation, and upregulated the expression of anti-aging factor Klotho.	[56]

## 5. Antioxidant Mechanism of Natural Antioxidant Peptides

After understanding the methods of evaluating antioxidant activity and the effects produced by these three types of methods, researchers aimed to explore the mechanisms behind the generation of these effects. In fact, the mechanism of antioxidant action lies in the effects of antioxidant peptides on redox signaling, and these effects lead to the expression or silencing of genes and proteins, resulting in a series of biological changes.

### 5.1. Regulation of Redox Signal Transduction Pathways and Gene Expression

**Figure 5:**
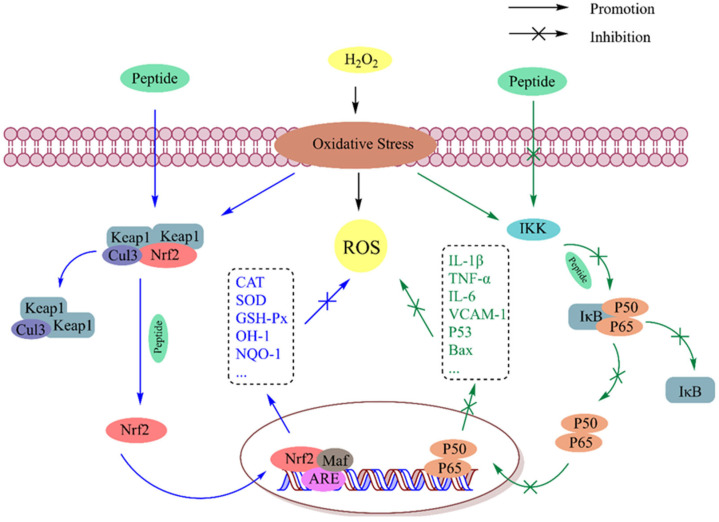
Regulation of the Nrf2 and NF-κB pathways by antioxidant peptides. Keap1: Kelch-like ECH-related protein 1; Cul3: cullin 3; Maf: oncogene homolog of myoaponeurotic fibrosarcoma; ARE: antioxidant response elements; IKK: inhibitor of kappa B kinase; IκB: NF-kappa-B inhibitor alpha; p50/p65: heterodimers, dubbed NF-κB.

ROS generated by oxidative stress trigger redox signaling by activating or repressing redox-sensitive transcription factors, thereby affecting protein function and leading to changes in signal output, enzyme activity, gene transcription, and membrane and genome integrity [57]. Nuclear factor E2-associated protein 2 (Nrf-2) and nuclear factor kappa B (NF-κB) are two transcription factors associated with redox control. Nrf2 encodes antioxidant and general cytoprotective genes, while NF-κB regulates the expression of proinflammatory genes. There is growing evidence that oxidative stress is closely related to the inflammatory response. Oxidative stress induces chronic inflammation, and the inflammatory response improves ROS release; this process mediated mainly by the NF-κB signaling pathway. Antioxidant peptides can regulate the transcription of multiple antioxidant genes by inhibiting NF-κB signaling and activating the Nrf2 pathway, thereby maintaining cellular homeostasis (Figure 5). The mechanisms of various antioxidant peptides are summarized in Table 6.

#### 5.1.1. Nrf2 Signaling Pathway

The Keap1-Nrf2-ARE (Kelch-like ECH-related protein 1-nuclear factor E2-related factor-antioxidant response element) signaling pathway is one of the most important cellular defense mechanisms that maintains physiological homeostasis, including in response to stress and inflammation. Under normal physiological conditions, most Nrf2 is sequestered in cytoplasmic lysates by the actin-binding inhibitor Keap1 and subsequently degraded via ubiquitination. In redox stress conditions, Nrf2 is released from Keap1 and translocates from the cytoplasm to the nucleus to heterodimerize with the small Maf (oncogene homolog of myoaponeurotic fibrosarcoma) protein. By recognizing the ARE in the promoter regions of target genes, the Nrf2-Maf complex activates the ARE-dependent gene expression of a range of antioxidant, phase II detoxification, and cytoprotective proteins, including genes encoding GST, GSH-Px, GR, CAT, SOD, NQO-1, HO-1, and thioredoxin reductase (TRXR) [58]. As previously shown, bioactive peptides can destroy the interaction of Keap1 and release free Nrf2 by occupying the active site of Keap1-Nrf2 and thus activating this pathway. Most of the current studies on antioxidant peptides focus on the activation of this pathway, and the potential molecular mechanisms of antioxidant peptides can be preliminarily predicted based on this pathway together with the use of molecular docking. Zhang et al. [59] explored the potential mechanism of antioxidant stress for walnut peptides in HT22 cells through molecular docking. The docking results showed that all six peptides could successfully bind to Keap1, and EYWNR and FQLPR could interact with the binding sites of Nrf2 in the Keap1-Kelch domain through hydrogen bonds to block the entrance to the cavity of their active sites, resulting in the overexpression of antioxidant enzymes in cells. The mechanism of peptide activation in this pathway can be further explored and verified at the gene and protein levels by using a combination of molecular docking and Western blotting. By investigating the molecular mechanism of the antioxidant tripeptide Pro-His-Pro in Chinese baijiu, Wu et al. [60] found that this peptide upregulated the mRNA and protein expression levels of antioxidant enzymes (CAT, SOD, and GSH-Px), increased the content of Nrf2, and decreased the levels of the Keap1 protein, thereby stimulating Nrf2-mediated ARE transcription. Through Western blotting and molecular docking, Ren et al. [61] found that the antioxidant peptide of crushed rice (SGDWSDIGGR) interacted with Keap1 and occupied the Nrf2 binding site of the Nrf2-Keap1 complex, which promoted the expression of Nrf2 in the nucleus and signaling pathway; as a result, the expression of HO-1 and SOD1 was regulated downstream of the Nrf2 pathway. The rice protein hydrolysate improved the oxidative state in mouse livers by upregulating the mRNA expression of CAT, SOD, and GSH-Px at the gene level, thus regulating the Keap1-Nrf2-ARE pathway; and the expression of NOX4, which is related to ROS synthesis, was inhibited [48].

#### 5.1.2. NF-κB Signaling Pathway

The NF-κB transcription factor is a key gene expression factor involved in the regulation of inflammation, the immune response, cell adhesion, and apoptosis. This factor plays a central role in the expression of genes encoding cytokines (such as tumor necrosis factor-α (TNF-α), interleukin-1β (IL-1β), IL-6 and IL-8, vascular cell adhesion molecule-1 (VCAM-1), and apoptosis protein (Bax)) [62]. The Rel/NF-κB inducible transcription factor family consists of five structure-related peptides that bind to form transcriptional homodimers or heterodimers, among which the p50/p65 heterodimer (known as NF-κB) is the most abundant bioactive dimer. These peptides consist of an approximately 300-amino-acid-long Rel homogenic region (RHR), and this RHR allows subunits to dimerize, recognize NF-κB, and bind to DNA. Under normal physiological conditions, NF-κB binds to the inhibitor IκB (Ikappa B) protein and stably localizes to the cytoplasmic complex. IκB protein kinase (IKK) can be activated during oxidative stress (in which NF-κB inducers become involved, including bacterial and viral products, inflammatory cytokines, ROS, and ultraviolet rays), and IκB protein is released by the IKK body; as a result, NF-κB is rapidly translocated to the nucleus and specific gene promoters are bound to activate the transcription and expression of specific proinflammatory and apoptotic genes [63]. A sturgeon muscle peptide (F2) purified by Gao et al. exhibited strong antioxidant activity and significantly inhibited the release of proinflammatory cytokines (IL-1β, IL-6, and TNF-α) by suppressing the expression of IκBα and NF-κB, suggesting that this antioxidant peptide could exert antioxidant effects by inhibiting the NF-κB pathway [64]. The nuclear protein p53 can bind to specific DNA sequences and activate NF-κB transcription. The wheat germ antioxidant peptide RVF mentioned above prevents the inhibitor IκB protein from being degraded or phosphorylated to resist NF-κB activation, leading to decreased expression of p53 and Bax [65]. Tong et al. [66] reported that the rice peptide AAGALPS inhibited the translocation of p65 from the cytoplasm to the nucleus and significantly inhibited the increase in IKK and degradation of IκB; in addition, the expression of the nuclear p65 protein and VCAM-1 was inhibited, indicating that rice peptide can improve the oxidative stress and inflammation of cells by inhibiting the NF-κB pathway. Chen et al. [67] discovered that the snake venom peptide SVP-1 (Tyr-Thr-Trp-Glu-Ala) significantly attenuated the overproduction of ROS and the overexpression of inflammatory cytokines (IL-6, IL-1β, and TNF-α) and thus inhibited the activation of the NF-κB signaling pathway.

#### 5.1.3. Other Signaling Pathways

Related studies have also reported the mechanisms of antioxidant peptides in nonmammals. The transcription factor DAF-16 in the *C. elegans* nematode is a direct homolog of the mammalian FOXO protein, and its downstream target gene is *SOD-3* [68], which participates in antioxidant defense, anti-stress responses, and metabolism. Zhao et al. [68] studied the action mechanism of antioxidant peptides in *Strongylocentrotus nudus* via RNA interference (RNAi). The researchers found that SnP7 (AAVPSGASTGIYEALELR) and SnP10 (NPLLEAFGNAK) could reduce the ROS content in the nematodes, promote DAF-16 nuclear translocation, and induce the expression of its target gene, *SOD-3*. Yu et al. [69] identified two antioxidant peptides, SeP2 (DVEDLEAGLAK) and SeP5 (EITSLAPSTM), that were produced from *Sepia esculenta*. They found that both of them could reduce ROS and MDA levels, increase SOD activity, and upregulate the expression of *SOD-3* in oxidatively damaged nematodes, and SeP5 could also upregulate the expression of *CAT-1*. These findings indicated that these two peptides could upregulate the expression of target genes to increase antioxidant stress by activating related signaling pathways. Wu et al. [70] studied sea cucumber peptides (SCPs) from *Acaudina leucoprocta* by using a *C. elegans* model and reported that these SCPs could increase the activity of SOD and CAT, decrease the content of MDA, and upregulate the expression of SOD-3 by regulating the DAF-16 signaling pathway, which enhances the antioxidant capacity of nematodes.

**Table 6:** Action mechanisms of natural antioxidant peptides from different sources.

Source	Peptide/Protein Hydrolysate	Oxidation Model	Antioxidant Mechanism	Reference
Walnut	EYWNR, FQLPR	HT22 cells exposed to H_2_O_2_	(1) The peptides reduced NF-κB and IκBα expression by preventing the phosphorylation and degradation of IκBα, thereby inhibiting the expression of pro-inflammatory factors in the NF-κB pathway	[59]
Chinese Baijiu	PHP	HepG2 cells exposed to AAPH	(1) The peptide inhibited the production of ROS, MDA, and GSSG, prevented the decrease in GSH, and upregulated the activity of antioxidant enzymes (2) It upregulated the mRNA and protein expression levels of antioxidant enzymes (CAT, SOD, and GSH-Px), increased the expression of Nrf2 protein, and decreased the levels of Keap1 protein, thereby stimulating the Keap1-Nrf2-ARE pathway.	[60]
Crushed rice	SGDWSDIGGR	Human embryonic lung diploid fibroblasts (2BS) cells exposed to H_2_O_2_	(1) This peptide entered the binding pocket of Keap1 and activated the Keap1-Nrf2 pathway through hydrogen bonding, thus increasing the protein expression of antioxidant enzymes.	[61]
Sturgeon muscle	F2 fraction	RAW264.7 cells exposed to LPS	(1) These peptides reduced NF-κB and IκBα expression by preventing the phosphorylation and degradation of IκBα, thereby inhibiting the expression of pro-inflammatory factors in the NF-κB pathway	[64]
Wheat germ	RVF	SH-SY5Y cells exposed to H_2_O_2_	(1) This peptide reduced the release of LDH and inhibited the accumulation of reactive oxygen species (2) It stabilized the inhibitor I κ B protein and decreased the protein expression of p53 and Bax, thus inhibiting the NF- κ B pathway.	[65]
Rice	AAGALPS	HUVECs exposed to TNF- α	(1) This peptide led to an increase in GSH-Px content, a decrease in MDA content, and the inhibition of ROS production (2) This peptide induced an increase in IKK and a corresponding inhibition of IκB degradation and nuclear p65 protein expression, leading to an inhibition of the NF-κB pathway.	[66]
Rice	Soluble rice protein hydrolysate	Oxidative stress in t-BHP-treated mice	(1) These peptides upregulated the mRNA expression of CAT, SOD, and GSH-Px, thus regulating the Keap1-Nrf2-ARE pathway	[48]
*Strongylocentrotus nudus*	AAVPSGASTGIYEALELR, NPLLEAFGNAK	Oxidative stress in paraquat-treated *C. elegans*	(1) These peptides reduced the content of ROS (2) They promoted the nuclear translocation of DAF-16 and induced the expression of target gene *SOD-3*.	[68]
*Sepia esculenta*	DVEDLEAGLAK, EITSLAPSTM	Oxidative stress in paraquat-treated *C. elegans*	(1) The peptides decreased the levels of ROS and MDA and increased the activity of SOD (2) They upregulated the expression of *SOD-3* or *CAT-1*	[69]
*Acaudina leucoprocta*	Protein hydrolysate	Oxidative stress in paraquat-treated *C. elegans*	(1) The peptides increased the activity of GSH-Px and SOD and decreased the activity of MDA and lipid peroxidation (2) They increased the activity of AChE, thus improving memory ability.	[70]

## 6. Structure-Activity Relationship

Most existing studies have focused on the isolation and purification of novel natural antioxidant peptides, and as a result, the mechanisms underlying the relationship between activity and structure and function are unclear. However, clarifying the structure-activity relationship of bioactive peptides is the best prerequisite for selecting enzymes with which to digest natural proteins and obtain antioxidant peptides more efficiently and specifically via enzymatic hydrolysis. Moreover, the antioxidant capacity of peptides can be predicted by analyzing their structure-activity relationships. Molecular weight, amino acid composition and sequence, and secondary structure are the key factors that affect antioxidant activity.

### 6.1. Molecular Weight

Most natural antioxidant peptides contain more than two amino acid residues and are mainly composed of between 2 and 10 amino acids, and their molecular weight is less than 1000 Da. Soybean peptides were extracted from fermented soybean meal and separated into four fractions, <1, 1–3, 3–5, and >5 kDa, via ultrafiltration. The scavenging rate of the <1 kDa peptide for DPPH radicals was the highest [46]. The molecular weights of the 12 major antioxidant peptides in Antarctic krill (*Euphausia superba*) mentioned above range from 327.34 to 603.68 Da, and the number of amino acid disabilities ranges from three to five [42]. Fan et al. [71] identified four novel antioxidant peptides from the fermented extract of duck liver, and these peptides consisted of 6–9 amino acids and had a relative molecular weight of between 723.45 and 981.51 Da. Xia et al. [72] identified eight novel antioxidant peptides from mung bean proteolytic digests, and these peptides consisted of 2–5 amino acids and had molecular weights of between 275 and 709 Da. A total of 31 peptides were identified from the peptide hydrolysate of grass carp scale gelatin, 24 of which were small-molecular-weight peptides with molecular weights within the range of 358–986 Da. Additionally, through chemical and cellular experiments, it was found that small-molecular-weight peptides showed high antioxidant activity in terms of scavenging ROS as well as regulating the intracellular antioxidant system [31,41]. Peptides with low molecular weights (<1 kDa) can significantly improve antioxidant activity, possibly because low-molecular-weight peptides act as electron donors to react with free radicals and block chain reactions; in addition, these peptides are more easily transported across the gastrointestinal barrier to the target site for utilization and absorption, thus increasing their bioavailability [73].

### 6.2. Amino Acid Composition and Sequence

The primary structure of a peptide refers to the order of amino acids on the peptide chain and the positions of disulfide bonds, which determine the overall chemical properties and biological activity of peptides. The amino acids that affect antioxidant activity can be classified according to their physical and chemical properties, including aromatic amino acids, hydrophobic amino acids, sulfur-containing amino acids, and acidic or basic amino acids. These amino acids and their action mechanisms are summarized in Table 7. Chen et al. [74] identified the antioxidant peptide FF-6 (Phe-Pro-Leu-Pro-Ser-Phe), which has hydrophobic or aromatic amino acid residues other than Ser, in *Allium tuberosum* Rottler. This peptide showed ultrahigh ABTS radical scavenging activity (94% scavenging rate) and oxygen free radical scavenging activity (ORAC = 13.8 ± 0.83 μ MTE/μM peptide), but almost no metal-ion-chelating activity was observed (for which the chelating rate of Fe^2+^ was less than 10%). Subsequent experiments indicated that Leu was the key site for scavenging free radicals, and the reason for the weak chelating ability of Fe^2+^ is the lack of acidic or basic amino acids. Lu et al. [75] reported that SYPTECRMR from sesame protein hydrolysate exhibited the highest antioxidant activity and confirmed that its active site is related to two sulfur-containing amino acids (Cys and Met); in addition, compared to the methylthio group of Met, the sulfhydryl group of Cys more easily provides electrons to electrophilic reagents, so the antioxidant activity of Cys is greater than that of Met. Among the five antioxidant peptides identified from Channa argus soup, P13, which lacks net charge residues (acidic amino acids and basic amino acids), exhibited the lowest Fe^2+^-chelating ability [31]. Among the soluble rice proteins with antioxidant and hepatoprotective effects, Glu (216.4 mg/g), Asp (104.5 mg/g), and Arg (86.0 mg/g) had relatively high content [48].

The sequence and positions of amino acids in polypeptides are important factors determining antioxidant activity. Wu et al. [76] designed a series of modified peptide sequences (including amino acid residue replacements or peptide truncations) based on the antioxidant peptide PMRGGGGYHY, demonstrating that peptide sequences play a fundamental role in antioxidant function. Cheng et al. [77] used alanine (Ala) to replace Leu, Phe, Tyr, and Pro in the antioxidant peptides LR5 (LHKFR) and YR6 (YGLYPR), respectively, demonstrating that amino acid sequences affect the activity of antioxidant peptides. According to Nwachukwu et al., the properties of amino acids located at the N-terminal or C-terminal positions in a sequence affect the antioxidant activity of a peptide [78]. Wang et al. [79] reported that walnut peptides containing hydrophobic amino acids at the N-terminus or C-terminus, such as QGRPWG, PSRADIY, and AYNIPVNIAR, exhibited strong antioxidant activity. The N-terminus or C-terminus of three novel antioxidant peptides (LSPGEL, VYFDR, and PGPTY) derived from *Gracilariopsis lemaneiformis* protein also contain hydrophobic amino acids [25]. Uno et al. [80] revealed that the presence of aromatic amino acids at the C-terminus or the hydrophobicity of N-terminal residues could significantly enhance antioxidant activity. Li et al. [81] reported that antioxidant activity is related to the electronic properties, hydrophobicity, hydrogen bonds, and spatial potential resistance of N-terminal and C-terminal amino acids, and the properties of C-terminal amino acids are more important than those of N-terminal amino acids.

### 6.3. Secondary Structure

The effects of amino acid composition and sequence on antioxidant activity were analyzed, revealing that the nature of and interaction between the side chain groups of amino acid residues affect the antioxidant activity of the corresponding peptide. However, the secondary structure of a protein (including α-helix, β-fold, β-rotation, and random crimp) is the spatial conformation of the peptide main chain and does not involve the side chain of amino acid residues. Compared with the composition and sequence of amino acids, the secondary structures of peptides play a more important role in antioxidative activity. As previously reported, Pro has a unique ring structure and a fixed ψ angle, which can change the secondary structure of peptides and the position and steric hindrance of active hydrogen atoms by affecting adjacent amino acids, thus affecting the antioxidant activity of peptides [82]. The peptides PGMLGGSPPGLGGSPP and SDGSNIHFPN with β-turns, β-folds, or α-helices identified in Channa argus soup exhibited relatively high antioxidant activity, while the DPPH radical scavenging activity of the randomly crimped SVSIRADGGEGEVTVFT was relatively low [31]. Zhang et al. [83] used a pulsed electric field (PEF) to treat the shrimp peptide QMDDQ to change its secondary structure. It was found that a PEF could transform β tablets into random curls and thus reduce the steric hindrance of free radicals. As a result, the modified peptide could scavenge free radicals more effectively, and the antioxidant activity of the peptides was improved. The secondary structure can affect the interaction between antioxidant peptides and free radicals by exposing or hiding the active sites of antioxidant peptides. However, the effects of secondary structures on antioxidative activity are complex and diverse, and the specific effects and mechanisms of different secondary structures should be further discussed in the future.

### 6.4. Computer Modeling-Assisted Analysis of the Structure-Activity Relationships of Natural Antioxidant Peptides

With the further development of bioactive peptide research and the cross-application of computer science and bioinformatics science, computer modeling-assisted analysis (CMAS) methods have been developed to help us determine the structure-activity relationships of natural antioxidant peptides more thoroughly. According to the existing research, four computer-assisted analysis methods, namely, molecular docking, quantum chemical calculations, and quantitative structure-activity relationship (QSAR) and molecular dynamics (MD) simulations, have been applied to study bioactive peptides.

#### 6.4.1. Molecular Docking

Molecular docking is a powerful tool that simulates interactions between molecules (such as receptors and ligands) and their relative spatial positions. This method provides rapid, efficient, and accurate information on the structure-activity relationships of antioxidant peptides by analyzing the interactions between antioxidant peptides and target proteins or small-molecule ligands and predicting their binding modes and binding abilities. Agrawal et al. [84] used molecular docking to reveal that free radicals and active amino acid residues play an antioxidant role by forming hydrogen bonds and engaging in hydrophobic and electrostatic interactions. Through molecular docking, Huang et al. [85] reported that the amino acid residues of SPSSS, SGTAV, and NSVAA could form hydrogen bonds or engage in hydrophobic interactions with free radicals.

#### 6.4.2. Quantum Chemistry Calculations

Quantum chemistry calculations based on density functional theory (DFT) can be used to effectively calculate the structure and activity of molecules via conformational optimization, highest occupied molecular orbital (HOMO) calculations, lowest unoccupied molecular orbital (LUMO) energy studies, and natural bond orbital (NBO) analysis. He et al. [86] performed a HOMO analysis and showed that the active sites of the peptides AFWYGLPCKL, GCFRYACGAFY, and WPWQMSLY were N29-H10 (on the indolyl group of Trp-3), O122-H38 (on the phenol group of Tyr-5), and serine and leucine, respectively.

#### 6.4.3. Quantitative Structure-Activity Relationship

QSAR modeling is a mathematical method used to quantitatively describe the relationship between the chemical structure and the biological activity of a studied compound and can be performed by determining the mathematical expression of the relationship between structure and activity. Tian et al. [87] established a QSAR model to analyze the structure-activity relationship of β-lactoglobulin antioxidant tripeptides and reported that the electrical and hydrogen-bonding properties of amino acids in the peptide sequence, as well as the spatial characteristics of C-terminal and N-terminal amino acid residues, play important roles in antioxidation. Zheng et al. [82] analyzed the structure-activity relationship of antioxidant dipeptides using QSAR and reported that Tyr, Trp, Cys, and Met play a leading role in antioxidant dipeptides. Dipeptides containing Tyr and Trp, especially Tyr/Trp residues at the N-terminus, are the most effective free radical scavengers and exhibit strong antioxidant activity, and the activity of adjacent residues is affected by space effects, hydrophobicity, and hydrogen bonding. These methods combined with experiments can further optimize the performance of natural antioxidant peptides and provide a direction for the subsequent synthesis of peptides with better antioxidant activity.

#### 6.4.4. Molecular Dynamics Simulation

Molecular dynamics simulation is a potent theoretical tool for investigating the structures and dynamics of peptides, with various force fields such as CHARMM, AM-BER, OPLS, and GROMOS having been developed for simulating biological macromolecules. Its application in predicting and analyzing antioxidant peptide structures has been demonstrated [88]. For instance, the docking of donkey skin gelatin peptide onto Keap-1 was simulated by molecular docking and MD, which indicated that the PGPAP peptide could bind to Keap1 in a strained and stable structure, with key binding sites identified on Arg415, Gly462, Phe478, and Tyr572 [89]. Additionally, researchers utilized 50 ns MD simulations to predict the binding interactions and protein-ligand stability of the synthetic antioxidant peptide Syn-Ake with matrix metalloproteinase (MMP) and Sirtuin 1 (SIRT1). The results showed that the Syn-Ake peptide remained stable at the active sites of MMP-13 and SIRT1 receptors and had anti-aging effects [90]. Furthermore, Zhang et al. employed a pulsed electric field to transform the β-folded structure in VNAVLH pine nut peptide into a randomly curled one, which enhanced its antioxidant activity; this was further elucidated through structure-activity relationship studies using MD simulations [91].

## 7. Safety Assessment, Bioavailability, and Application of Antioxidant Peptides

### 7.1. Safety Evaluation of Antioxidant Peptides

Although natural antioxidant peptides are generally considered nontoxic, their potential allergenicity and toxicity should be verified prior to consumption when used in functional foods. Zheng et al. [92] confirmed the lack of toxicity of the coconut cake globulin antioxidant peptide YILIK using computer techniques. The antioxidant peptide Lys-Gly-Phe-Arg from *Decapterus maruadsi* was judged to be nontoxic using ToxinPred (http://crdd.osdd.net/raghava/toxinpred/index.html) accessed on 18 August 2019 [12]. Most low-molecular-weight natural peptides do not produce allergic reactions but may contain allergenic sequences, as predicted by Marco et al. [93] using computer tools like ToxinPred and AllerTOP (http://www.ddg-pharmfac.net/AllerTOP) accessed on 14 February 2019. However, these studies are predictive, and how to systematically conduct experimental studies to assess the safety of bioactive peptides is an important quandary that we will continue to research in the future.

### 7.2. Bioavailability of Antioxidant Peptides

The bioavailability of antioxidant peptides has been previously validated in vitro and in vivo, demonstrating certain biological effects. The ability of antioxidant peptides to remain active in vivo suggests resistance to modification and degradation by gastrointestinal proteases. Peptide transport across epithelial cells into the bloodstream occurs through four modes: (1) carrier-mediated transport (PepT1 transporter protein), (2) the paracellular pathway, (3) transcytosis, and (4) passive diffusion [94,95]. Caco-2 cells, forming a monolayer with tight connections, contain enzymes capable of degrading and metabolizing short peptides, resulting in the excretion of safe and non-toxic peptides. The amino acid composition and sequence may change after absorption and excretion. Therefore, simulation of gastrointestinal digestion using human intestinal Caco-2 epithelial cells in vitro is a widely recognized method for testing peptide stability and permeability to predict bioavailability.

Antioxidant peptides (Gly-Pro-Phe, Gly-Pro-Glu, and Phe-Gly-Glu) extracted from wheat germ were found to be well-absorbed through passive transport across the intestinal epithelium [96]. Simulation of the digestive process revealed that soy peptides exhibited free radical scavenging capacity positively correlated with protein and glycine concentrations [97]. *Dendrobium aphyllum*-derived antioxidant peptides with molecular weights lower than 1 kDa showed high uptake characteristics; however, their free amino acid compositions and sequences were altered upon excretion by Caco-2 cells [98]. Moreover, Ser-Ser-Arg could pass through the Caco-2 monolayer membrane and was considered to have high bioavailability. To verify the gastrointestinal stability of casein antioxidant peptides, Xie et al. [99] simulated two processes: gastrointestinal digestion and Caco-2 cell uptake. The peptide fractions below 1000 Da showed poor structural stability in the first phase and excellent antioxidant stability throughout all phases, and three peptides with stable antioxidant properties in vivo were identified: Ile-Glu, Ser-Asp-Lys, and Ala-Tyr-Pro-Ser. The casein-derived peptide LHSMK was found to have good antioxidant activity when taken up through Caco-2 cell monolayers [100]. Furthermore, it was demonstrated that specific antioxidant peptides could be transported via different pathways such as paracellular or PepT1-mediated transport using a Caco-2 cell monolayer model as well as animal experiments [101].

The aforementioned evidence indicates that natural antioxidant peptides can be absorbed into the bloodstream through various transport pathways, thus exhibiting a certain degree of bioavailability. However, most current studies have focused on the activity and mechanisms of antioxidant peptides, with limited attention paid to their absorption, metabolism, and antioxidant processes.

### 7.3. Current Applications of Antioxidant Peptides

In terms of current applications, the research and development of natural antioxidant peptides have primarily centered around three areas. Firstly, in the food industry, natural antioxidant peptides can enhance the antioxidant properties of foods while also providing nutritional benefits. This has led to their use in functional foods, nutritional and health products, and dietary supplements. For example, low-molecular-weight antioxidant peptides (<3 kDa) obtained from mackerel gelatine were found suitable for fortifying carbonated beverages due to their solubility and emulsifying activity [102]. Lentil protein hydrolysate with high antioxidant activity was shown to improve the antioxidant properties of baked goods without affecting their organoleptic properties or hardness [103]. Amaranth hydrolyzed proteins were demonstrated to be able to enhance the quality of bread, namely, sourdough, by improving its organoleptic properties and shelf life [104].

Secondly, natural antioxidant peptides have the ability to alleviate oxidative stress and inflammation by modulating redox signaling. In the field of pharmaceuticals, these peptides can serve as pharmaceutical ingredients and therapeutic agents for treating diseases associated with oxidative damage and inflammation. Previous cellular experiments have demonstrated that antioxidant peptides are effective in treating oxidative damage and inflammation, as well as in improving memory, reducing blood pressure, and extending lifespan. These peptides can be applied in production for these purposes. Furthermore, monkfish (*Lophius litulon*) peptides have been found to modulate gut flora imbalance and kidney injury induced by oxidative stress and inflammation, thereby ameliorating nephrotoxicity caused by a high-fat diet [105]. Additionally, it has been shown that egg white peptides promote healthy cell migration during wound healing, increase serum SOD and CAT activities, and accelerate the healing of mechanically damaged skin wounds at both the cellular and animal levels [106].

Thirdly, regarding the field of cosmetics, natural antioxidant peptides such as glutathione and carnosine are widely utilized bioactive peptides in skincare products. Prolonged exposure to UV radiation can lead to structural changes in the skin, including collagen fiber shortening and thickening, elastin fiber damage, loss of type I collagen, and alterations in dermal collagen composition [107]. Studies have demonstrated that antioxidant peptides (<3.5 kDa) derived from skipjack tuna (Katsuwonus pelamis) significantly protect human skin fibroblasts (HSFBs) from UVA-induced damage and could potentially be incorporated into cosmetic formulations [108]. Furthermore, numerous studies have indicated that collagen peptides possess the ability to mitigate photoaging by scavenging excess free radicals within the body, enhancing antioxidant enzyme activity, and reducing oxidative stress to counteract free radical-induced damage [107]. Wang et al. [109] also demonstrated that hen-derived collagen peptides attenuated UVA-induced damage in human dermal fibroblasts (HDF). It is worth noting that the applications of antioxidant peptides extend far beyond these examples, with ongoing extensive research expected to yield further discoveries in this area.

## 8. Conclusions

Natural antioxidant peptides have been widely studied for their ability to ameliorate and prevent conditions and diseases caused by oxidative stress. Moreover, natural antioxidant peptides can be used as medicinal ingredients in cosmetics to alleviate skin aging, reduce melanin accumulation, and regulate collagen. However, research on natural antioxidant peptides in various fields remains limited, and their commercialization faces many challenges. Furthermore, additional research is needed to fill gaps in our knowledge of antioxidant peptides in terms of their in vivo stability, biological relevance, and safety. This paper summarizes progress in research on the application of natural antioxidant peptides to evaluate activity, structure-activity relationships, and action mechanisms in recent years. Natural antioxidant peptides have potential applications in nutritional and health products, cosmetics, and therapeutic agents. With the deepening of related research and the interdisciplinary application of computer science, statistics, animal science, and clinical medicine, the long-term goal of the commercial application of natural antioxidant peptides will become a reality.

## Figures and Tables

**Table 3:** Chemical evaluation methods in vitro and their biological effects from different antioxidant peptides.

Source	Peptide	Method	Measured Activity	Reference
Corn	RYLL	DPPH and ABTS radical scavenging activity	Scavenging free radicals	[34]
*Ganoderma lucidum*	DRVSIYGWG, ALLSISSF	DPPH and ABTS radical scavenging activity	Scavenging free radicals	[13]
Watermelon seeds	RDPEER, KELEEK, DAAGRLQE, LDDDGRL, GFAGDAPRA	DPPH and ABTS radical scavenging activity, ORAC, Fe^2+^ chelating ability	Scavenging free radicals; chelating metal ions	[15]
Feathers	LPGPILSSFPQ	ABTS and ^•^OH radical scavenging activity, Fe^2+^ chelating ability, TRAP, FRAP, TBARS	Scavenging free radicals; chelating metal ions; inhibition of lipid peroxidation	[23]
Chicken	RWGG, YYCQ	ABTS radical scavenging activity, ORAC	Scavenging free radicals	[24]
Milk	YVPR, VPYPQR	DPPH and ABTS radical scavenging activity, CBA	Scavenging free radicals	[25]
*Gracilaria lemaneiformis*	LSPGEL, VYFDR, PGPTY	DPPH and ABTS radical scavenging activity, FRAP	Scavenging free radicals	[26]
Yak casein	RELEEL	DPPH, O_2_^•−^ and ^•^OH radical scavenging activity, FRAP, Fe^2+^ chelating ability	Scavenging free radicals; chelating metal ions	[28]
Pea	YLVN, EEHLCFR, TFY	DPPH, ABTS, O_2_^•−^ and ^•^OH radical scavenging activity, ORAC	Scavenging free radicals	[35]
Shrimp meat	MTTNL, MTTNI	Measurement of scavenging free radicals of DPPH and ^•^OH via electron paramagnetic resonance spectroscopy	Scavenging free radicals	[26,36]
*Channa* *argus*	IVLPDEGK, PGMLGGSPPGLLGGSP, SDGSNIHFPN, SVSIRADGGEGEVTVFT.	DPPH and ^•^OH radical scavenging activity, Fe^2+^ chelating ability	Scavenging free radicals; chelating metal ions	[31]

**Table 7:** Effects of amino acid composition on antioxidant activities.

Types of Amino Acids	Names	Action Mechanism	Reference
Aromatic amino acids	Tyr, Trp, Phe, His	These amino acids can act as hydrogen donors (providing protons to electron-deficient free radicals) and improve ROS scavenging capacity.	[74]
Hydrophobic amino acids	Val, Leu, Tyr, Trp, Phe, Ile, Pro, Met	These amino acids can act as hydrogen donors and inhibit lipid peroxidation by enhancing the solubility of peptides in the lipid phase and improving the accessibility of fat-soluble ROS or polyunsaturated fatty acids.	[67]
Sulfur-containing amino acids	Cys, Met	These amino acids provide electrons to scavenge ROS and can be converted into GSH, and Met can be oxidized to methionine sulfoxide and has antioxidant activity.	[75]
Acidic amino acids	Glu, Asp	These amino acids have carboxyl and amino side chains that chelate with metal ions through electrostatic interactions.	[31,48]
Basic amino acids	His, Arg, Lys		

Note: valine: Val; leucine: Leu; isoleucine: Ile; phenylalanine: Phe; tryptophan: Trp; methionine: Met; proline: Pro; glycine: Gly; serine: Ser; cysteine: Cys; tyrosine: Tyr; histidine: His; lysine: Lys; arginine: Arg; aspartic acid: Asp; glutamic acid: Glu.
